# Supplementary material for: The value of time in the invigoration of human movements when interacting with a robotic exoskeleton
Source: Sci Adv. 2023 Sep 20;9(38):eadh9533. doi: 10.1126/sciadv.adh9533 (PMC10511201; doi:10.1126/sciadv.adh9533)
Supplement: Supplementary file 1 — Supplementary Text Figs. S1 and S2 Tables S1 to S11 [file sciadv.adh9533_sm.pdf]

Science Advances
AAAS

Supplementary Materials for

The value of time in the invigoration of human movements when interacting with a robotic exoskeleton

Dorian Verdel *et al.*

Corresponding author: Dorian Verdel, dorian.verdel@ens-paris-saclay.fr

Sci. Adv. **9**, eadh9533 (2023)
DOI: 10.1126/sciadv.adh9533

This PDF file includes:

Supplementary Text
Figs. S1 and S2
Tables S1 to S11

The value of time in the invigoration of human movements when interacting with a robotic exoskeleton: Supplementary Materials

1 Average velocity profiles small amplitude

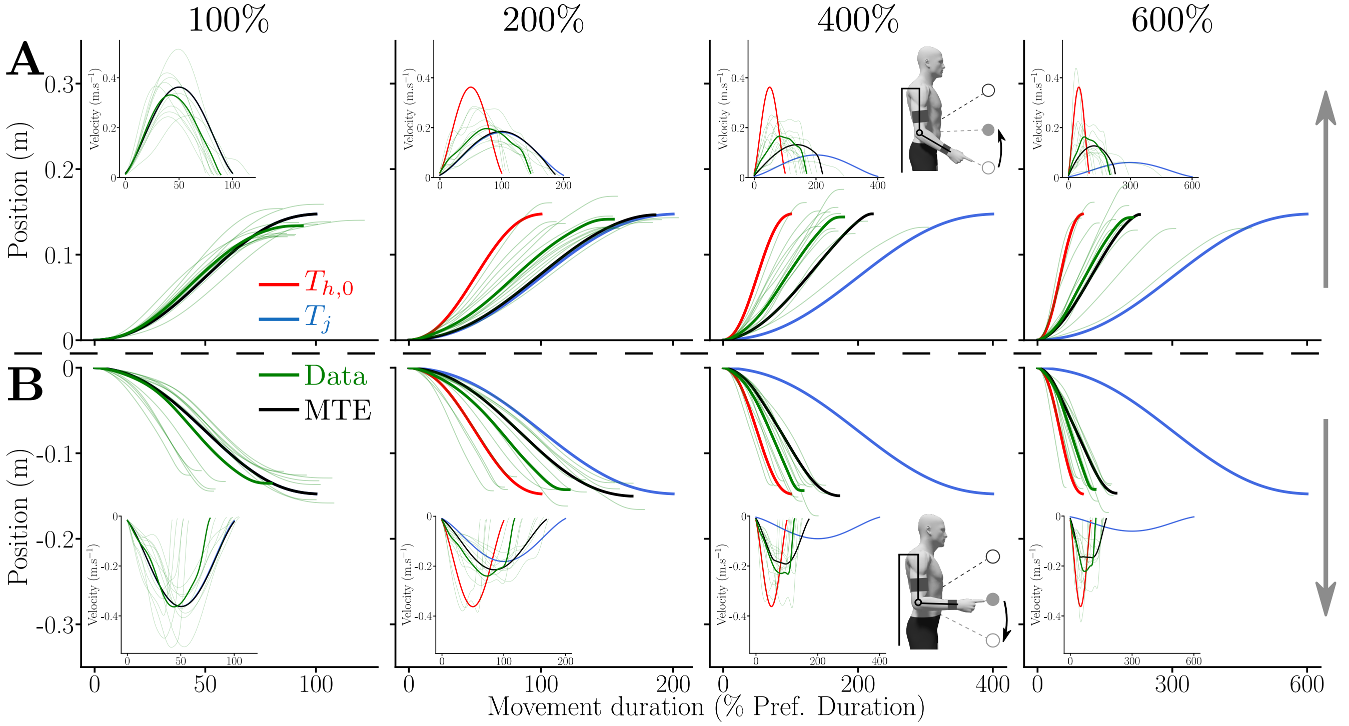

Figure S.1: **Average trajectories measured for the small amplitude (SA) and for each assistance duration.** In green the average recorded position and velocity profiles (as insets), in light green the individual trajectories, in black the MTE predictions, in blue the minimum jerk planned by the assistance and in red the constant time strategy. In the 100% condition, the red and blue curves are covered by the black curve. **A**. Upward movements. **B**. Downward movements.

2 Consistency of inter-individual differences in the *test* session

Since vigor is known to be an idiosyncratic parameter [31], a natural question arising when analysing the impact of time on human movement regards the inter-individual differences and the ability to predict them. Previous works have shown the ability of the minimum time-effort (MTE) theory to predict these inter-individual differences accurately, when the CoT is identified as in the present paper [35]. However, before trying to predict these differences, it was necessary to assess whether they are present and consistent as in previous works. In contrast to the previous work [35], the current protocol was normalized both in terms of time and effort, with respect

to each participant's nominal characteristics. The impact of such a normalization on inter-individual differences was quantified with a linear mixed model to assess a potential effect of vigor on the participant's behavior during the *test* session.

The linear mixed model analysis was performed on the three main parameters describing the participants' behavior. Only upward movements were considered in the analysis because participants were mostly passive during downward movements. The significance of this test was set at $p < 0.05$ with a Bonferroni-Holm correction. The model accounted for fixed effects of amplitude, condition and movement vigor with random effects depending on the participant. Finally, the model was as in Equation S.1 (using Wilkinson's notation):

$$P_i \sim \text{amplitude} + \text{amplitude}:\text{condition} + \text{vigor} + (1|\text{subject}) \tag{S.1}$$

where P_i was the predicted parameter (*i.e.*, MD in $\%$ Preferred Duration, maximum relative interaction force in $\%\text{F}^{\text{Flex}}_{\text{max}}$ or work in J). The results of these analyses highlighted a significant contribution of the amplitude term to the relative duration and interaction force (in both cases: $p < 10^{-15}$) and of the $\text{amplitude}:\text{condition}$ term to all the tested parameters (in all cases: $p < 10^{-20}$). Importantly, no significant effect of the participant's vigor in predicting MD, relative maximum interaction force and work was found ($p = 1, p = 1$ and $p = 0.18$ respectively). Since the p value was relatively low for the work, we considered that further analyses might reveal an effect of vigor on this parameter. Consequently we performed Pearson correlation analyses between the work and vigor of participants for each amplitude and condition separately, with a Bonferroni-Holm correction as previously. However, these analyses did not return any significant result (in all cases: $r < 0.66$ and $p > 0.18$). Finally, it can be concluded that the normalization of the protocol was effective, which resulted in a participants' behavior independent of their nominal vigor in the task.

3 Methodology for the identification of the CoT

To test the minimum time-effort model, it was necessary to identify the CoT given a model of effort in order to replicate the nominal vigor of the average participant. This problem had been addressed in previous studies [29,31,35,46], and a composite cost function additioning time and effort terms was considered as follows:

$$C(\mathbf{u}, T) = \int_0^T \ell(\mathbf{x}(t), \mathbf{u}(t))dt + \int_0^T g(t)dt \tag{S.2}$$

where $\mathbf{u}$ is the control variable, T is the duration of movement, $\mathbf{x}$ is the state vector, $\ell(\mathbf{x}, \mathbf{u})$ is the infinitesimal cost of movement (*i.e.*, effort) and $g(t)$ is the infinitesimal cost of time, the integral of which is the true CoT.

In practice, the CoT was estimated by simulating trajectories of various amplitudes A in fixed time $T = T(A)$, based on the average amplitude-duration relationship measured during the baseline experiment (pooling all participants and movement directions). It has been demonstrated that the infinitesimal value of the CoT at time T is equal to the opposite value of the optimal Hamiltonian $\mathcal{H}_0$ associated with the problem in fixed time T [29], as follows:

$$g(T) = -\mathcal{H}_0\left(\mathbf{x}(T), \mathbf{p}(T), \mathbf{u}(T), \lambda = 1\right) \tag{S.3}$$

where $\mathbf{p}$ is the unique optimal co-state vector verifying Pontryagin's Maximum Principle with $\lambda = 1$ [85,86], and $\mathbf{x}$, $\mathbf{u}$ are respectively the optimal state and optimal control of the fixed-time optimal control problem. More precisely, the Hamiltonian was computed as follows:

$$\mathcal{H}_0\left(\mathbf{x}(T), \mathbf{p}(T), \mathbf{u}(T), \lambda = 1\right) = \mathbf{p}^\top(T)\mathbf{f}(\mathbf{x}(T), \mathbf{u}(T)) + \lambda\ell(\mathbf{x}(T), \mathbf{u}(T)) \tag{S.4}$$

where $\mathbf{f}(\mathbf{x}, \mathbf{u}) = \dot{\mathbf{x}}$ represents the system dynamics in state space. By varying the amplitude A, different values of the infinitesimal CoT $g(T)$, can be obtained and these data points revealed a sigmoidal shape as in previous works [29,31]. This resulted in the fitted curves presented in Figure S.2.

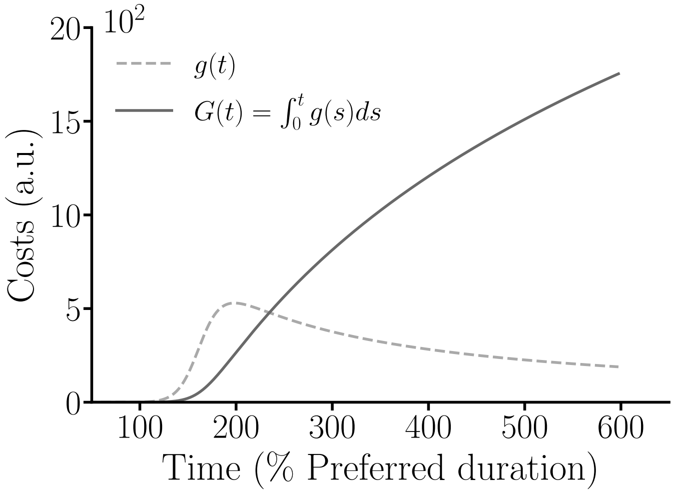

Figure S.2: **Illustration of the CoT obtained in the present study.** The curve $G(t)$ is the CoT used in the simulations to predict optimal movement durations from the MTE for the new situations created by the exoskeleton. This sigmoidal shape was deduced from the values $g(t)$ obtained during the identification procedure.

4 Individual data

All data introduced in Tables S.1–S.11 are presentted under the form: $\text{average} \pm \text{std.dev}$.

Participant	MD_{A0} (s)	MD_{A1} (s)	MD_{A2} (s)	MD_{A3} (s)
S1	0.522 ± 0.091	0.706 ± 0.130	1.080 ± 0.370	1.239 ± 0.604
S2	0.748 ± 0.212	0.931 ± 0.220	1.265 ± 0.428	1.700 ± 0.264
S3	0.573 ± 0.134	0.650 ± 0.083	0.726 ± 0.081	0.809 ± 0.260
S4	0.625 ± 0.120	1.066 ± 0.174	1.227 ± 0.170	1.444 ± 0.209
S5	0.723 ± 0.276	0.773 ± 0.108	0.944 ± 0.272	1.028 ± 0.288
S6	0.475 ± 0.074	0.622 ± 0.102	0.690 ± 0.156	1.085 ± 0.534
S7	0.497 ± 0.065	0.730 ± 0.110	0.836 ± 0.145	1.023 ± 0.290
S8	0.609 ± 0.169	0.772 ± 0.089	0.866 ± 0.120	1.116 ± 0.130
S9	0.558 ± 0.083	0.845 ± 0.213	1.103 ± 0.286	1.491 ± 0.268
S10	0.616 ± 0.010	0.905 ± 0.198	1.116 ± 0.258	1.478 ± 0.163
S11	0.538 ± 0.048	0.774 ± 0.061	0.857 ± 0.053	1.031 ± 0.194
S12	0.449 ± 0.062	0.568 ± 0.067	0.675 ± 0.084	0.696 ± 0.183

Table S.1: **Movement durations (MD) for upward movements during the *baseline* experiment.** The indices $\{A0, A1, A2, A3\}$ correspond to the four tested amplitudes of the *baseline* experiment (*i.e.*, $\{8.75°, 17.5°, 26.25°, 35°\}$).

Participant	MD_{A0} (s)	MD_{A1} (s)	MD_{A2} (s)	MD_{A3} (s)
S1	0.587 ± 0.123	0.877 ± 0.207	1.184 ± 0.551	1.416 ± 1.112
S2	0.992 ± 0.260	1.303 ± 0.298	1.427 ± 0.413	1.734 ± 0.457
S3	0.581 ± 0.113	0.672 ± 0.102	0.807 ± 0.091	0.823 ± 0.133
S4	0.788 ± 0.157	1.132 ± 0.154	1.258 ± 0.153	1.329 ± 0.193
S5	0.614 ± 0.102	0.798 ± 0.196	1.015 ± 0.235	1.183 ± 0.144
S6	0.612 ± 0.151	0.713 ± 0.113	0.769 ± 0.132	0.930 ± 0.083
S7	0.626 ± 0.071	0.715 ± 0.122	0.841 ± 0.201	1.041 ± 0.285
S8	0.653 ± 0.104	0.823 ± 0.170	1.034 ± 0.177	1.004 ± 0.100
S9	0.673 ± 0.087	0.887 ± 0.164	1.082 ± 0.248	1.223 ± 0.280
S10	0.754 ± 0.171	0.917 ± 0.113	1.112 ± 0.143	1.443 ± 0.170
S11	0.633 ± 0.049	0.814 ± 0.067	0.993 ± 0.124	1.092 ± 0.080
S12	0.500 ± 0.050	0.573 ± 0.032	0.668 ± 0.089	0.857 ± 0.157

Table S.2: **Movement durations (MD) for downward movements during the *baseline* experiment.** The indices $\{A0, A1, A2, A3\}$ correspond to the four tested amplitudes of the *baseline* experiment (*i.e.*, $\{8.75°, 17.5°, 26.25°, 35°\}$).

Participant	Vigor Up (a.u.)	Vigor Down (a.u.)
S1	0.970	0.897
S2	0.743	0.683
S3	1.306	1.307
S4	0.798	0.829
S5	1.035	1.023
S6	1.205	1.242
S7	1.134	1.156
S8	1.049	1.066
S9	0.857	0.960
S10	0.839	0.874
S11	1.101	1.053
S12	1.496	1.431

Table S.3: **Vigor data obtained during the *baseline* experiment.** Separated scores were computed for upward movements and downward movements and for each participant.

Participant	Cond.	Amp.	MD (% Pref. Dur.)	Max. Force ($\%F_{max}^{Flex}$)	Work (J)
S1	100%	SA	100.738 ± 1.698	-6.166 ± 3.886	-0.508 ± 0.189
S1	100%	LA	87.355 ± 0.801	-6.010 ± 1.14	-1.122 ± 0.177
S2	100%	SA	106.629 ± 5.317	1.696 ± 1.048	0.355 ± 0.277
S2	100%	LA	78.075 ± 1.404	0.496 ± 0.658	-0.652 ± 0.598
S3	100%	SA	92.218 ± 7.054	3.357 ± 1.117	-0.029 ± 0.306
S3	100%	LA	95.928 ± 2.992	8.308 ± 2.445	1.830 ± 0.722
S4	100%	SA	82.766 ± 1.498	-1.525 ± 0.652	-0.654 ± 0.089
S4	100%	LA	84.140 ± 1.304	-2.774 ± 0.853	-2.384 ± 0.251
S5	100%	SA	78.754 ± 11.318	-1.376 ± 0.001	-0.334 ± 0.005
S5	100%	LA	83.877 ± 2.997	-3.177 ± 1.053	-2.297 ± 0.709
S6	100%	SA	121.783 ± 23.695	10.249 ± 0.924	-0.046 ± 0.404
S6	100%	LA	105.889 ± 18.381	11.094 ± 1.040	1.542 ± 1.453
S7	100%	SA	90.156 ± 4.881	-4.862 ± 1.917	-1.222 ± 0.269
S7	100%	LA	86.840 ± 1.182	-4.584 ± 0.369	-2.803 ± 0.237
S8	100%	SA	86.650 ± 3.238	0.604 ± 0.935	-0.178 ± 0.231
S8	100%	LA	80.955 ± 1.339	0.294 ± 0.931	-0.725 ± 0.655
S9	100%	SA	90.824 ± 2.382	3.144 ± 2.076	0.134 ± 0.223
S9	100%	LA	94.449 ± 19.500	-0.193 ± 1.884	-0.419 ± 0.349
S10	100%	SA	86.986 ± 3.764	0.421 ± 1.245	-0.115 ± 0.219
S10	100%	LA	82.357 ± 1.566	2.428 ± 1.612	0.419 ± 0.514
S11	100%	SA	84.701 ± 3.083	0.798 ± 1.288	-0.480 ± 0.300
S11	100%	LA	84.960 ± 4.293	1.499 ± 2.096	-0.625 ± 0.306
S12	100%	SA	103.321 ± 5.859	3.887 ± 1.596	0.860 ± 0.555
S12	100%	LA	96.088 ± 4.864	10.401 ± 1.887	4.933 ± 0.931

Table S.4: **Values of the three main studied parameters for upward movements for $T_j = 100\%$ of preferred movement duration.** Data are provided for both amplitudes.

Participant	Cond.	Amp.	MD (% Pref. Dur.)	Max. Force ($\%F_{max}^{Flex}$)	Work (J)
S1	200%	SA	184.264 ± 5.622	19.856 ± 9.857	1.061 ± 0.566
S1	200%	LA	182.285 ± 47.549	20.66 ± 21.674	1.823 ± 2.040
S2	200%	SA	191.198 ± 10.785	0.905 ± 0.696	0.088 ± 0.288
S2	200%	LA	146.237 ± 5.795	2.990 ± 1.646	1.371 ± 1.488
S3	200%	SA	154.303 ± 19.864	11.282 ± 0.872	1.432 ± 0.186
S3	200%	LA	144.990 ± 19.049	25.586 ± 4.131	6.580 ± 1.177
S4	200%	SA	158.817 ± 9.484	0.949 ± 0.781	0.035 ± 0.150
S4	200%	LA	158.598 ± 6.136	7.247 ± 2.520	3.283 ± 1.325
S5	200%	SA	155.722 ± 19.593	4.799 ± 2.969	0.699 ± 0.552
S5	200%	LA	156.239 ± 6.171	-0.924 ± 1.998	-1.730 ± 0.709
S6	200%	SA	204.015 ± 26.475	8.817 ± 1.306	0.265 ± 0.492
S6	200%	LA	184.908 ± 14.469	11.303 ± 1.827	3.209 ± 1.443
S7	200%	SA	111.358 ± 40.394	9.711 ± 7.396	1.486 ± 1.738
S7	200%	LA	158.694 ± 13.130	-2.775 ± 5.128	-2.223 ± 2.258
S8	200%	SA	152.376 ± 8.072	1.888 ± 1.020	0.158 ± 0.294
S8	200%	LA	123.003 ± 39.247	6.628 ± 1.331	4.505 ± 0.824
S9	200%	SA	159.406 ± 5.730	10.195 ± 1.665	1.029 ± 0.192
S9	200%	LA	151.606 ± 0.587	-1.286 ± 1.467	-0.657 ± 0.311
S10	200%	SA	118.238 ± 10.297	13.090 ± 0.494	2.237 ± 0.079
S10	200%	LA	144.987 ± 6.045	6.322 ± 4.217	1.538 ± 1.352
S11	200%	SA	108.140 ± 16.941	10.161 ± 1.529	2.418 ± 0.609
S11	200%	LA	131.175 ± 27.613	8.029 ± 2.550	3.684 ± 1.215
S12	200%	SA	157.978 ± 5.269	2.659 ± 0.859	0.528 ± 0.227
S12	200%	LA	120.799 ± 19.266	13.383 ± 2.373	5.994 ± 0.758

Table S.5: **Values of the three main studied parameters for upward movements for $T_j = 200\%$ of preferred movement duration.** Data are provided for both amplitudes.

Participant	Cond.	Amp.	MD (% Pref. Dur.)	Max. Force ($\%F_{max}^{Flex}$)	Work (J)
S1	400%	SA	213.871 ± 78.507	39.789 ± 11.822	2.331 ± 0.928
S1	400%	LA	311.231 ± 17.443	15.863 ± 10.856	0.805 ± 1.362
S2	400%	SA	304.939 ± 77.197	3.258 ± 1.214	0.917 ± 0.429
S2	400%	LA	226.810 ± 105.680	2.930 ± 1.086	0.896 ± 0.569
S3	400%	SA	144.923 ± 6.109	9.676 ± 1.331	0.882 ± 0.487
S3	400%	LA	130.952 ± 9.550	29.142 ± 0.878	8.051 ± 0.443
S4	400%	SA	165.881 ± 8.938	11.926 ± 0.431	2.517 ± 0.185
S4	400%	LA	179.144 ± 33.826	11.815 ± 2.193	5.280 ± 1.251
S5	400%	SA	143.950 ± 24.235	12.509 ± 1.260	2.443 ± 0.393
S5	400%	LA	160.437 ± 43.473	13.188 ± 4.115	5.164 ± 1.935
S6	400%	SA	170.230 ± 22.092	15.236 ± 1.084	2.502 ± 0.175
S6	400%	LA	153.193 ± 18.117	19.809 ± 0.940	6.691 ± 0.345
S7	400%	SA	158.517 ± 100.166	15.501 ± 8.806	2.866 ± 1.808
S7	400%	LA	287.573 ± 72.238	0.753 ± 9.566	-0.451 ± 4.579
S8	400%	SA	195.632 ± 46.252	5.447 ± 1.864	1.314 ± 0.656
S8	400%	LA	192.152 ± 37.478	7.535 ± 2.233	4.863 ± 1.649
S9	400%	SA	170.357 ± 18.392	20.562 ± 1.467	2.214 ± 0.145
S9	400%	LA	165.435 ± 44.570	20.958 ± 7.226	4.610 ± 1.752
S10	400%	SA	174.023 ± 67.749	14.336 ± 6.292	2.219 ± 1.035
S10	400%	LA	170.961 ± 54.860	14.972 ± 4.699	5.018 ± 1.769
S11	400%	SA	123.054 ± 11.795	12.978 ± 1.956	3.226 ± 0.412
S11	400%	LA	179.479 ± 36.828	9.064 ± 2.848	3.942 ± 1.986
S12	400%	SA	178.516 ± 9.600	13.053 ± 0.747	3.593 ± 0.228
S12	400%	LA	177.916 ± 6.800	17.804 ± 1.228	8.405 ± 0.603

Table S.6: **Values of the three main studied parameters for upward movements for $T_j = 400\%$ of preferred movement duration.** Data are provided for both amplitudes.

Participant	Cond.	Amp.	MD (% Pref. Dur.)	Max. Force ($\%F_{max}^{Flex}$)	Work (J)
S1	600%	SA	476.697 ± 107.463	20.186 ± 12.975	0.648 ± 0.863
S1	600%	LA	116.073 ± 13.614	50.379 ± 4.770	5.997 ± 0.445
S2	600%	SA	222.665 ± 36.692	8.412 ± 0.534	2.827 ± 0.153
S2	600%	LA	166.612 ± 12.713	9.341 ± 0.588	6.176 ± 0.385
S3	600%	SA	97.091 ± 5.100	4.151 ± 0.832	-0.550 ± 0.449
S3	600%	LA	140.212 ± 15.022	31.838 ± 2.519	9.305 ± 0.680
S4	600%	SA	173.484 ± 17.498	12.396 ± 0.672	2.696 ± 0.319
S4	600%	LA	123.775 ± 9.423	20.538 ± 3.243	9.401 ± 1.105
S5	600%	SA	137.251 ± 22.052	10.626 ± 6.881	2.309 ± 1.568
S5	600%	LA	210.664 ± 55.780	13.126 ± 2.242	4.856 ± 1.518
S6	600%	SA	203.022 ± 30.786	16.458 ± 1.398	2.662 ± 0.223
S6	600%	LA	203.227 ± 58.663	16.412 ± 1.206	5.704 ± 0.341
S7	600%	SA	307.435 ± 171.620	6.746 ± 9.911	0.296 ± 2.369
S7	600%	LA	210.004 ± 126.348	14.564 ± 8.507	6.255 ± 3.144
S8	600%	SA	222.076 ± 104.645	8.483 ± 3.070	2.168 ± 1.321
S8	600%	LA	137.414 ± 38.890	13.441 ± 3.116	7.882 ± 0.597
S9	600%	SA	176.110 ± 21.481	23.012 ± 1.214	2.429 ± 0.250
S9	600%	LA	159.017 ± 43.546	23.568 ± 2.719	5.029 ± 0.540
S10	600%	SA	197.026 ± 38.440	16.352 ± 0.533	2.488 ± 0.177
S10	600%	LA	127.425 ± 59.714	21.301 ± 1.991	7.144 ± 0.626
S11	600%	SA	125.912 ± 10.837	13.884 ± 1.054	3.527 ± 0.297
S11	600%	LA	172.557 ± 10.866	13.224 ± 1.587	6.583 ± 0.880
S12	600%	SA	203.958 ± 20.186	14.404 ± 0.721	3.998 ± 0.321
S12	600%	LA	186.225 ± 11.825	21.215 ± 0.671	10.226 ± 0.391

Table S.7: **Values of the three main studied parameters for upward movements for $T_j = 600\%$ of preferred movement duration.** Data are provided for both amplitudes.

Participant	Cond.	Amp.	MD (% Pref. Dur.)	Max. Force ($\%F_{max}^{Ext}$)	Work (J)
S1	100%	SA	53.793 ± 24.753	-13.192 ± 2.332	0.328 ± 0.245
S1	100%	LA	72.397 ± 28.170	-17.497 ± 4.421	1.200 ± 0.203
S2	100%	SA	54.421 ± 10.339	-1.625 ± 0.961	-0.487 ± 0.197
S2	100%	LA	89.767 ± 11.651	-3.853 ± 0.851	1.299 ± 0.399
S3	100%	SA	101.876 ± 2.666	-17.738 ± 1.733	1.514 ± 0.200
S3	100%	LA	103.993 ± 1.739	-18.646 ± 2.205	4.132 ± 0.612
S4	100%	SA	43.190 ± 1.020	-6.327 ± 0.591	0.420 ± 0.074
S4	100%	LA	74.669 ± 23.031	-9.738 ± 0.898	1.660 ± 0.357
S5	100%	SA	107.207 ± 7.506	-2.068 ± 0.005	0.335 ± 0.005
S5	100%	LA	83.695 ± 20.986	-9.691 ± 1.998	1.217 ± 0.803
S6	100%	SA	101.303 ± 21.054	-6.195 ± 3.214	-0.170 ± 0.440
S6	100%	LA	93.912 ± 5.279	4.173 ± 2.221	-4.191 ± 0.532
S7	100%	SA	59.020 ± 16.717	-10.343 ± 1.059	1.318 ± 0.231
S7	100%	LA	67.597 ± 19.634	-9.950 ± 1.415	2.499 ± 0.111
S8	100%	SA	95.658 ± 1.662	-4.370 ± 0.570	0.559 ± 0.120
S8	100%	LA	85.243 ± 17.247	-6.010 ± 2.158	1.006 ± 0.369
S9	100%	SA	85.299 ± 24.054	-8.499 ± 1.124	0.215 ± 0.209
S9	100%	LA	86.800 ± 28.395	-7.843 ± 2.613	0.396 ± 0.649
S10	100%	SA	48.194 ± 7.579	-6.093 ± 1.317	0.116 ± 0.146
S10	100%	LA	55.538 ± 13.032	-4.040 ± 1.507	-0.549 ± 0.533
S11	100%	SA	100.047 ± 1.009	-5.615 ± 0.596	1.147 ± 0.080
S11	100%	LA	104.604 ± 8.125	-4.105 ± 0.514	-0.014 ± 0.074
S12	100%	SA	108.072 ± 4.122	-6.390 ± 0.864	1.183 ± 0.274
S12	100%	LA	95.310 ± 5.231	-7.308 ± 1.515	1.836 ± 0.520

Table S.8: **Values of the three main studied parameters for downward movements for $T_j = 100\%$ of preferred movement duration.** Data are provided for both amplitudes.

Participant	Cond.	Amp.	MD (% Pref. Dur.)	Max. Force ($\%F_{max}^{Ext}$)	Work (J)
S1	200%	SA	63.810 ± 8.191	-6.220 ± 2.721	-0.086 ± 0.171
S1	200%	LA	65.509 ± 6.033	-12.904 ± 3.255	0.627 ± 0.224
S2	200%	SA	74.575 ± 6.561	-2.928 ± 0.987	0.066 ± 0.230
S2	200%	LA	111.264 ± 29.033	-1.371 ± 1.660	-0.792 ± 0.544
S3	200%	SA	134.1700 ± 39.102	-22.280 ± 5.030	2.146 ± 0.560
S3	200%	LA	131.334 ± 45.359	-29.038 ± 8.408	6.038 ± 2.248
S4	200%	SA	81.536 ± 8.089	-6.791 ± 0.969	-0.168 ± 0.095
S4	200%	LA	119.913 ± 29.513	-4.963 ± 1.668	0.093 ± 0.676
S5	200%	SA	111.981 ± 19.534	-11.130 ± 2.505	0.394 ± 0.365
S5	200%	LA	108.473 ± 23.967	-7.919 ± 2.644	0.289 ± 0.717
S6	200%	SA	169.645 ± 20.131	-7.974 ± 1.255	0.277 ± 0.208
S6	200%	LA	180.617 ± 7.692	0.001 ± 5.199	-1.635 ± 1.269
S7	200%	SA	72.372 ± 10.140	-12.051 ± 3.759	2.034 ± 0.791
S7	200%	LA	91.543 ± 9.699	-8.442 ± 0.653	2.582 ± 0.250
S8	200%	SA	133.712 ± 33.264	-4.973 ± 0.500	0.529 ± 0.144
S8	200%	LA	139.273 ± 36.461	-2.043 ± 0.783	0.101 ± 0.501
S9	200%	SA	155.599 ± 38.002	-10.499 ± 1.983	0.512 ± 0.155
S9	200%	LA	76.546 ± 6.436	-12.945 ± 1.285	0.928 ± 0.121
S10	200%	SA	159.537 ± 34.035	-9.445 ± 2.356	0.591 ± 0.369
S10	200%	LA	83.061 ± 9.687	-4.343 ± 0.943	0.266 ± 0.172
S11	200%	SA	120.131 ± 42.440	-10.964 ± 3.520	2.138 ± 0.762
S11	200%	LA	130.156 ± 35.661	-8.661 ± 2.255	2.801 ± 1.488
S12	200%	SA	178.142 ± 3.719	-13.295 ± 0.698	2.678 ± 0.263
S12	200%	LA	113.922 ± 28.092	-12.173 ± 3.238	4.313 ± 1.584

Table S.9: **Values of the three main studied parameters for downward movements for $T_j = 200\%$ of preferred movement duration.** Data are provided for both amplitudes.

Participant	Cond.	Amp.	MD (% Pref. Dur.)	Max. Force ($\%F_{max}^{Ext}$)	Work (J)
S1	400%	SA	80.081 ± 11.940	-4.294 ± 2.231	-0.429 ± 0.208
S1	400%	LA	60.809 ± 9.254	-14.866 ± 4.319	1.073 ± 0.382
S2	400%	SA	110.869 ± 15.944	-2.846 ± 0.971	0.047 ± 0.265
S2	400%	LA	155.313 ± 11.882	-3.076 ± 0.823	-0.340 ± 0.482
S3	400%	SA	155.574 ± 5.404	-27.101 ± 2.836	2.579 ± 0.257
S3	400%	LA	108.992 ± 8.749	-17.734 ± 4.202	3.620 ± 0.496
S4	400%	SA	121.496 ± 17.770	-14.812 ± 2.281	-0.215 ± 0.211
S4	400%	LA	154.458 ± 9.273	-8.739 ± 1.307	0.077 ± 0.158
S5	400%	SA	131.716 ± 17.034	-11.995 ± 2.561	0.476 ± 0.220
S5	400%	LA	142.397 ± 22.410	-7.762 ± 4.187	0.754 ± 1.359
S6	400%	SA	152.132 ± 51.680	-6.345 ± 3.057	0.044 ± 0.906
S6	400%	LA	152.771 ± 34.280	0.756 ± 2.021	-0.955 ± 0.483
S7	400%	SA	72.940 ± 11.078	-12.790 ± 6.650	2.339 ± 1.321
S7	400%	LA	117.002 ± 3.285	-8.784 ± 2.528	3.178 ± 0.841
S8	400%	SA	135.870 ± 27.906	-5.702 ± 1.178	0.568 ± 0.269
S8	400%	LA	122.705 ± 64.929	-3.301 ± 0.996	0.889 ± 0.796
S9	400%	SA	90.394 ± 11.343	-7.723 ± 1.316	-0.091 ± 0.081
S9	400%	LA	108.136 ± 19.953	-6.232 ± 3.114	-0.005 ± 0.486
S10	400%	SA	113.521 ± 22.209	-4.420 ± 2.985	-0.038 ± 0.311
S10	400%	LA	181.677 ± 47.935	-5.304 ± 2.613	-0.569 ± 0.976
S11	400%	SA	117.012 ± 8.571	-5.974 ± 1.243	0.986 ± 0.273
S11	400%	LA	161.823 ± 9.085	-4.431 ± 1.491	0.979 ± 0.558
S12	400%	SA	140.848 ± 7.271	-9.350 ± 0.942	1.757 ± 0.219
S12	400%	LA	127.139 ± 2.988	-12.361 ± 0.770	4.705 ± 0.388

Table S.10: **Values of the three main studied parameters for downward movements for $T_j = 400\%$ of preferred movement duration.** Data are provided for both amplitudes.

Participant	Cond.	Amp.	MD (% Pref. Dur.)	Max. Force ($\%F_{max}^{Ext}$)	Work (J)
S1	600%	SA	63.347 ± 17.262	-8.248 ± 3.496	0.059 ± 0.299
S1	600%	LA	81.426 ± 10.815	-3.805 ± 4.175	-0.247 ± 0.422
S2	600%	SA	168.291 ± 17.788	0.836 ± 0.816	-0.578 ± 0.115
S2	600%	LA	181.765 ± 14.721	-0.739 ± 3.311	-0.559 ± 0.327
S3	600%	SA	101.119 ± 2.818	-22.723 ± 2.003	1.689 ± 0.305
S3	600%	LA	106.832 ± 12.691	-29.812 ± 11.366	4.606 ± 0.473
S4	600%	SA	112.975 ± 9.944	-18.817 ± 2.174	0.084 ± 0.153
S4	600%	LA	164.775 ± 12.484	-9.979 ± 6.403	-0.941 ± 0.267
S5	600%	SA	158.836 ± 60.107	-18.832 ± 22.463	1.102 ± 2.227
S5	600%	LA	161.850 ± 22.785	-6.828 ± 1.935	0.281 ± 0.552
S6	600%	SA	165.017 ± 23.984	-1.056 ± 2.108	-0.286 ± 0.190
S6	600%	LA	211.231 ± 67.747	-0.131 ± 4.972	-0.790 ± 1.088
S7	600%	SA	88.352 ± 16.100	-9.147 ± 1.619	1.493 ± 0.302
S7	600%	LA	133.959 ± 15.739	-8.352 ± 1.914	3.020 ± 0.612
S8	600%	SA	131.491 ± 17.753	-5.934 ± 1.247	0.771 ± 0.305
S8	600%	LA	190.874 ± 54.114	-11.432 ± 13.112	2.943 ± 1.515
S9	600%	SA	94.687 ± 25.156	-7.221 ± 0.914	-0.087 ± 0.159
S9	600%	LA	96.941 ± 22.088	-8.943 ± 5.640	0.418 ± 0.565
S10	600%	SA	162.356 ± 31.616	-2.163 ± 2.145	-0.462 ± 0.183
S10	600%	LA	178.733 ± 24.195	0.751 ± 2.266	-0.881 ± 0.654
S11	600%	SA	134.227 ± 10.438	-6.926 ± 0.865	1.051 ± 0.220
S11	600%	LA	167.635 ± 14.959	-6.871 ± 3.297	1.804 ± 0.602
S12	600%	SA	157.591 ± 18.241	-10.602 ± 1.603	2.093 ± 0.379
S12	600%	LA	132.000 ± 7.572	-16.175 ± 4.248	5.697 ± 0.502

Table S.11: **Values of the three main studied parameters for downward movements for $T_j = 600\%$ of preferred movement duration.** Data are provided for both amplitudes.
